# MyFitnessPal smartphone application: relative validity and intercoder reliability among dietitians in assessing energy and macronutrient intakes of selected Filipino adults with obesity

**DOI:** 10.1136/bmjnph-2023-000770

**Published:** 2024-02-02

**Authors:** Mary Grace Banal, Demetria Bongga, Jose Maria Angbengco, Sophia Amarra, Leonora Panlasigui

**Affiliations:** 1 School of Nutrition, Philippine Women's University, Manila, Philippines; 2 Department of Food Science and Nutrition, University of the Philippines Diliman College of Home Economics, Quezon City, Philippines; 3 St. Luke's Medical Center, Quezon City, Philippines; 4 University of the Philippines Diliman College of Home Economics, Quezon City, Philippines

**Keywords:** Nutrition assessment, Weight management, Preventive counselling

## Abstract

This study aimed to explore the validity of energy and macronutrient intake estimates provided by a popular nutrition tracking smartphone application. 37 obese Filipino adults and 3 nutritionist–dietitians participated in this study. Participants used MyFitnessPal to log their food intake for 5 days. They also completed paper-based food record forms at the same time. Dietitians then referred to each of the participants’ completed food record forms to log the participants’ food intakes and generated estimates of energy and nutrient intake using the same app. The researcher also referred to the participants’ completed food record forms and generated energy and nutrient intake data using the Food Composition Tables (FCT)—the Philippine reference standard for estimating calorie and nutrient intakes. T-tests showed no statistical difference in energy and macronutrient data generated between participants and dietitians using MyFitnessPal app but Bland-Altman plots showed very weak to moderate agreements. T-tests revealed statistically significant difference between using the MyFitnessPal app and FCT in estimating energy, protein and fat intakes and Bland-Altman plots showed very weak to moderate agreement between MyFitnessPal and FCT. MyFitnessPal was found to underestimate the values for energy, carbohydrates and fat and overestimate values for protein when compared with estimates using FCT. Analysis of variance showed good intercoder reliability among dietitians, with the exception of fat intake estimates. The Goldberg approach showed very low likelihood of misreporting energy intake among the participants in this study. In this study, MyFitnessPal showed poor validity among Filipinos with obesity but with good reliability when used by dietitians. It also showed poor validity relative to the FCT. Prior nutrition knowledge is a factor in ensuring the accuracy of energy and nutrient intake data generated using MyFitnessPal app. It is recommended that users consult with dietitians for guidance on how to use these apps in weight management interventions.

**WHAT IS ALREADY KNOWN ON THIS TOPIC**

- Nutrition tracking apps enable users to estimate their energy and macronutrient intake without relying on nutritionist–dietitians.

**WHAT THIS STUDY ADDS**

- Results of this study showed MyFitnessPal to have poor construct validity and poor relative validity among Filipinos with obesity but with good intercoder reliability among dietitians.

**HOW THIS STUDY MIGHT AFFECT RESEARCH, PRACTICE, OR POLICY**

- Results of this study suggest that this MyFitnessPal may be better used as an adjunct to conventional interventions or low-intensity approaches rather than as intensive stand-alone interventions.

## Introduction

Apps such as MyFitnessPal enable users to log the food and beverages they eat and, consequently, estimate their energy and macronutrient intake without relying on nutritionist–dietitians. These apps have been studied in different countries and can potentially help individuals with obesity with socioeconomic restrictions in self-monitoring their food and nutrient intake as part of behavioural approach to weight management.^1–5^

Accuracy and reliability of these apps, however, should be considered when nutrition interventions are based on the assessment of dietary intake of individuals.^6^ Unfortunately, these apps currently lack scientific validation especially since these apps are developed and studied within the context of Western societies and other developed countries.^7^ Due to the lack of clarity regarding the accuracy of information nutrition apps provide with regards to dietary assessment and limited data regarding their applicability among local users, Filipinos with obesity and Filipino healthcare professionals may lack confidence over mobile health applications in nutrition assessments and obesity management.

Hence, the purpose of this study was to determine the validity of using the smartphone app MyFitnessPal as a dietary assessment tool as well as the reliability among the intended users of this app. Specifically, it aims to determine the: (1) level of agreement of caloric and macronutrient intake data using MyFitnessPal between participants and dietitians, and (2) level of agreement between caloric and macronutrient intake data provided by MyFitnessPal smartphone application and data computed using the standard method of dietary assessment among individuals with obesity; and (3) level of agreement of caloric and macronutrient intake data generated using MyFitnessPal among nutritionist–dietitians. The following are the hypotheses for this study:

### Construct validity

H_0_: there is no statistically significant difference between mean intakes of calorie and macronutrients reported using MyFitnessPal between the participants and dietitians.

### Relative validity

H_0_: there is a no statistically significant difference between mean intakes of calorie and macronutrients reported between the FCT and MyFitnessPal.

### Intercoder reliability:

H_0_: there is no statistically significant difference among mean intakes of calorie and macronutrients computed using MyFitnessPal among nutritionist–dietitians.

## Methods

This is a cross-sectional validation study conducted in Manila, Philippines from April to June 2022. A sample size of 35 participants was determined based on convenience and a similar validation study^3^ and to account for possible dropouts during the study. Additionally, a convenience sample of five dietitians was needed for this study. Participation to this study was solicited through social media and online communities catering to Filipinos with obesity and Filipino dietitians. Eligibility requirements include body mass index (BMI) of ≥28 kg/m^2^, age 20–40 years, at least high school graduate, own a smartphone, have basic computer skills and internet access. Height and weight measurements were self-reported following detailed instructions provided by researcher on how to accurately measure and record height and weight at home. Dietitian participants must own a smartphone and have a stable internet and agree to use the MyFitnessPal app. Due to COVID-19 restrictions, all interactions were conducted online and limited to Greater Manila Area residents with reliable internet.

Participants in the study used both MyFitnessPal^8^ and paper-based food records^9^ to record their dietary intake for five consecutive days. The paper-based food records included descriptions of food, meal times and amounts consumed in household measures or grams. To prevent memory bias, they were instructed to use MyFitnessPal every meal and to use the paper-based food diary at the end of the day. Additionally, participants completed the International Physical Activity Questionnaire (IPAQ).^10 11^ Participants were advised not to alter their normal diet and physical activity for the study. Participants attended an online workshop where they were taught by the researcher how to use the app and encode portion size information using standard measuring cups/spoons or food weighing scale. They then exported their energy and nutrient intake data from MyFitnessPal and sent it to the researcher via email. The participants also sent a digital copy of their food records to the researcher. From these food records, all five dietitians estimated the calorie and macronutrient intake of each of the participants using MyFitnessPal.

To estimate the nutrient intake of the participants using the local reference standard, the online Food Composition Tables (FCT) was used. When certain foods were not available in the FCT database, the researcher selected the nearest alternative from the database or conducted online searches for nutrition facts.

To determine the relative and construct validity of MyFitnessPal, Student’s t-test was used to assess associations^12^ and Bland-Altman plots were used to quantify agreement by analysing mean differences and limits of agreement.^13 14^ A p value<0.05 was considered statistically significant.^15^

To determine intercoder reliability of MyFitnessPal app, agreement of participants’ nutrient intake estimates using MyFitnessPal among dietitians was assessed using single factor analysis of variance (ANOVA).^16 17^

To validate self-reported dietary intake, subjects were categorised as acceptable reporters (AR), under-reporters (UR), or over-reporters (OR) based on their energy intake (EI) ratio to estimated energy expenditure (EE) using the Goldberg formula.^18 19^ In the Goldberg approach, subjects were identified as AR, UR, or OR from their ratio of EI based on the FCT calorie intake estimates to estimated EE according to whether the individual’s EI:EE ratio was within, below or above the 95% confidence limits of agreement between the two measurements.^18^ Predicted energy expenditure was based on the recommended EI per day by the Philippine Dietary Reference Intakes.^20^ Estimated EE was the product of physical activity level (PAL) and Basal Metabolic Rate (BMR). PAL was estimated from the IPAQ.^10 11^ BMR was estimated for each participant using Harris-Benedict Energy Equation.^21^

## Results

A total of 37 out of 41 participants completed the study. Mean age is 23.9±3.7 years. Seventy-six per cent were females and 9% were males. BMI was 32.0±3.9 kg/m^2^. Most of the participants were college students (54%), and the rest were either college graduate (43%) or high school graduate (3%). Five registered nutritionist–dietitians were recruited for this study. Four participants and two dietitians were not able to complete the required data within the specified time and chose to withdraw from the study.

### Construct validity: comparison of energy and nutrient intake assessment using MyFitnessPal between participants and dietitians

Only the data sets submitted by the three dietitians who were able to compute the energy and macronutrient intakes of all the participants using MyFitnessPal app were included in the data analysis. Table 1 presents the descriptive statistics for energy and nutrient intake estimates and results of the t-test used to calculate the association of energy and macronutrient intake using MyFitnessPal between participants and dietitians.

**Table 1:** Statistical difference between participants’ and dietitians’ MyFitnessPal data (n=37)

Variable	Participant group	Descriptive statistics		T-test		
		Mean	SD	P value (T≤t) two-tail	T critical two-tail	Decision
Energy (kcal)	Participants	1702	700.4	0.14	1.97	Do not reject Ho
	Dietitians	1804	58.3			
Carbohydrates (g)	Participants	215.6	101.5	0.23		Do not reject Ho
	Dietitians	227.9	10.8			
Protein (g)	Participants	76.9	40.8	0.19		Do not reject Ho
	Dietitians	72.2	2.8			
Fat (g)	Participants	54.5	28.1	0.03		Reject Ho
	Dietitians	61.1	6.8			

Except for fat, no statistically significant difference was found in mean intakes of energy and macronutrients between MyFitnessPal data from participants and dietitians. Except for protein, dietitians recorded higher intakes of energy and macronutrients compared with participants.

#### Limits of agreement between participants and dietitians

**Figure 1:**
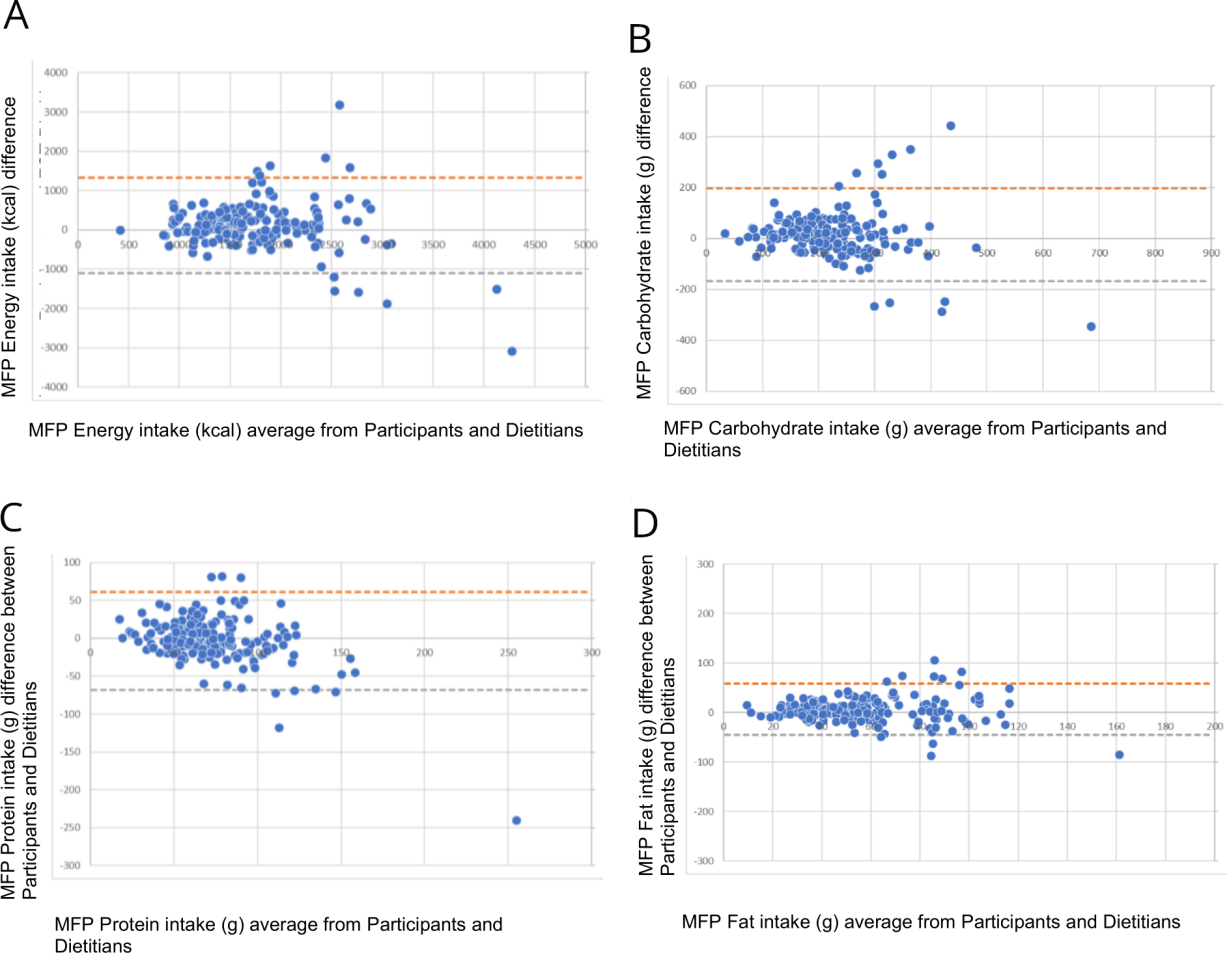
Bland-Altman plot for (A) energy (kcal), (B) carbohydrates (g), (C) protein (g) and (D) fat (g) intake data using MyFitnessPal (MFP) between participants and dietitians with 95% limits of agreement.

For energy, the mean difference was 111 kcal and the 95% limits of agreement were −1102 kcal to 1325 kcal. Agreement with random relative error suggests weak correlation. The mean difference of carbohydrates (g) was 14 g with limits of agreement from −169 g to 197 g. Agreement with random relative error suggests very weak correlation. Protein (g) had a mean difference of −4 g with 95% limits of agreement from −68 g to 61 g. Agreement with random relative error suggests moderate correlation. The mean difference of fat (g) was 6 g with 95% limits of agreement from −45 g to 58 g. Agreement with random relative error suggests very weak correlation (figure 1A–D).

### Relative validity: comparison of energy and nutrient intake between MyFitnessPal and FCT

Table 2 presents the mean estimated intakes of energy (kcal), carbohydrate (g), protein (g) and fat (g) as recorded by the participants using MyFitnessPal and the participants’ energy and macronutrient intakes as computed by the research assistant using the FCT. It also shows the t-test results used in calculating energy and macronutrient intake association between MyFitnessPal and FCT.

**Table 2:** Statistical difference between MyFitnessPal and food composition tables (FCT) (n=37)

Variable	Dietary assessment tool	Descriptive statistics		T-test		
		Mean	SD	P (T≤t) two-tail	T critical two-tail	Decision
Energy (kcal)	MyFitnessPal	1702.1	700.4	0.05	1.97	Reject Ho
	FCT	1848.1	697.2			
Carbohydrates (g)	MyFitnessPal	215.6	101.5	0.06		Do not reject Ho
	FCT	236.9	115.0			
Protein (g)	MyFitnessPal	76.9	40.8	0.00		Reject Ho
	FCT	61.5	24.0			
Fat (g)	MyFitnessPal	54.5	28.1	0.00		Reject Ho
	FCT	72.5	41.0			

Except for carbohydrates, statistically significant difference was found in mean intakes of energy and macronutrients between MyFitnessPal and FCT. Except for protein, MyFitnessPal recorded lower intakes of energy and macronutrients compared with FCT.

#### Limits of agreement between MyFitnessPal and FCT

**Figure 2:**
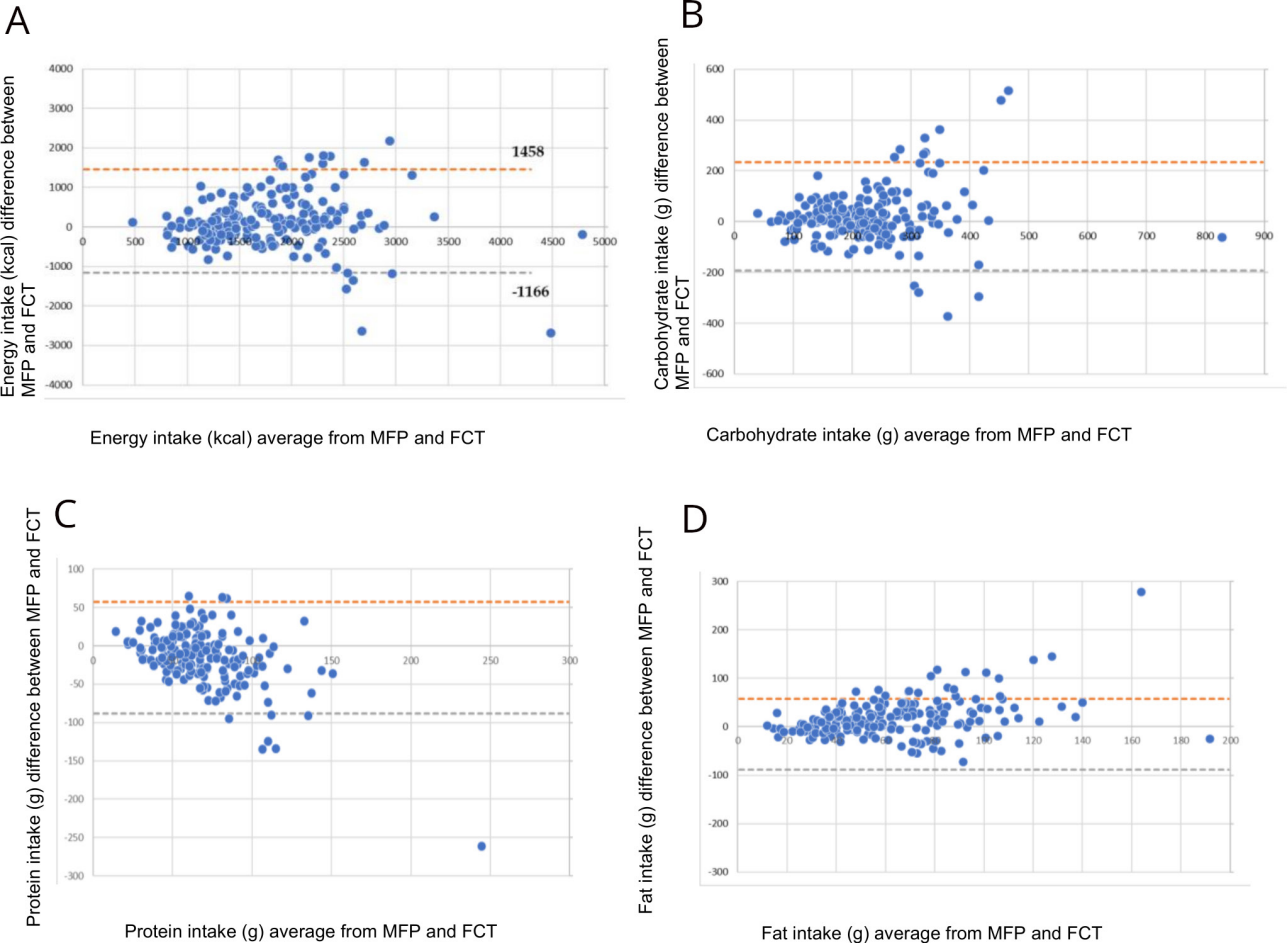
Bland-Altman plot for (A) energy (kcal), (B) carbohydrates (g), (C) protein (g) and (D) fat (g) from MyFitnessPal (MFP) and FCT with 95% limits of agreement. FCT, Food Composition Tables.

For energy, the mean difference was 146 kcal and the 95% limits of agreement were −1166 kcal to 1458 kcal. Agreement with random relative error suggests very weak correlation. The mean difference of carbohydrates (g) was 21 g with limits of agreement from −193 g to 235 g. Agreement with random relative error suggests very weak correlation. Protein (g) had a mean difference of −15 g with 95% limits of agreement from −88 g to 58 g. Agreement with random relative error suggests moderate correlation. For fat (g), the mean difference was 18 g with 95% limits of agreement from −60 g to 97 g. Agreement with random relative error suggests weak correlation (figure 2A–D).

### Intercoder reliability: comparison of energy and nutrient intake assessment using MyFitnessPal among dietitians

Table 3 presents the mean calculated intakes for energy (kcal), carbohydrate (g), protein (g), and fat (g) using MyFitnessPal among the three dietitians. It also shows the results of ANOVA used in determining statistically significant differences of the participants’ energy and macronutrient intakes using MyFitnessPal among dietitians

**Table 3:** Statistical difference of MyFitnessPal data among dietitians (n=3)

Variable	Dietitian	Descriptive statistics			ANOVA		
		Mean	SD	Median	P value	F crit	Decision
Energy (kcal)	Dietitian 1	1750.2	578.4	1685	0.35	3.013	Do not reject Ho
	Dietitian 2	1866.1	669.7	1789			
	Dietitian 3	1797.8	956.8	1635			
Carbohydrates (g)	Dietitian 1	239.2	106.4	223	0.14		Do not reject Ho
	Dietitian 2	226.6	96.4	217			
	Dietitian 3	217.8	99.1	199			
Protein (g)	Dietitian 1	70.1	28.6	66	0.27		Do not reject Ho
	Dietitian 2	75.3	31.3	72			
	Dietitian 3	71.0	35.4	64			
Fat (g)	Dietitian 1	54.0	26.1	50	0.001		Reject Ho
	Dietitian 2	67.6	38.5	59			
	Dietitian 3	61.7	37.5	54			

ANOVA, analysis of variance.

Except for fat, no statistically significant difference was found among dietitians when they computed the participants’ mean intakes of energy and macronutrients using MyFitnessPal. For fat, at least one batch mean is not equal to the others.

### Likelihood of misreporting energy intake

To screen out implausible energy intake (EI) reports, the Goldberg approach was used. Total energy expenditure (EE) ranged from 1487 kcal/day to 2920 kcal/day. PAL ranged from 1.2 (low) to 1.55 (high).

The CV_EI_ for EI recorded using the MyFitnessPal app and FCT was 41% and 38%, respectively. The coefficient of variation of EE was estimated from the PAL which was 35%. The 95% confidence limits of agreement between EI and EE were calculated as:

MyFitnessPal app:

$\pm 1SD=\sqrt{\frac{CV_{REI}^{2}}{5}+CV_{PER}^{2}+CV_{TEE}^{2}}\;=42\%$

FCT:

$\pm 1SD=\sqrt{\frac{CV_{REI}^{2}}{5}+CV_{PER}^{2}+CV_{TEE}^{2}}\;=41\%$

When using MyFitnessPal, two participants were classified as OR for 1 day only and one participant was classified as UR for 1 day only. Out of 185 days of reported intake, 182 days fell within the allowed range. Hence, at 95% CI, 98% of the total reported intakes were classified as acceptable reports of EI.

When FCT was used, only one participant was classified as OR for 1 day only and no participant was classified as UR. Out of 185 days reported intake, 184 days fell within the allowed range. Hence, at 95% CI, 99% of the total reported intakes were classified as acceptable reports of EI.

## Discussion

### Construct validity: comparison of energy and nutrient intake estimates using MyFitnessPal between participants and dietitians

Overall, the results of this study showed poor level of agreement between participants and dietitians in estimating energy and macronutrient intake using MyFitnessPal despite non-significant differences in energy and nutrient intake estimates. Findings show that the ability of MyFitnessPal to accurately estimate energy and macronutrient intakes of users is questionable.

Though relatively convenient, using nutrition apps in calorie tracking requires finding the time, tools such as food scales and measuring cups and spoons to correctly estimate portion sizes, and knowledge, not to mention skills, to employ all these correctly.^22^ This may help explain the weak agreement between the nutrient intake estimates generated using MyFitnessPal by the participants versus by nutrition professionals trained and experienced in dietary assessment. The use of standard dietary assessment tools such as FCT is often reserved to nutritionist–dietitians for they have the tools, knowledge, training and skills necessary to encode accurate information and correctly calculate for nutrient content of foods. This suggests that prior nutrition knowledge and skills play a role in the accuracy of nutrient data generated using nutrition apps such as MyFitnessPal.

### Relative validity: comparison of energy and nutrient intake between MyFitnessPal and standard dietary assessment tool

In this study, MyFitnessPal was found to underestimate the values for energy, carbohydrates and fat and overestimate values for protein. A similar study also showed that MyFitnessPal tends to underestimate ingestion of nutrients probably due to inadequacies in its database.^3^ In terms of volume of food composition database (FCD) data, consumer-grade apps actually have greater data volume (14 million foods claimed by developers of MyFitnessPal) compared with standard food databases which usually contain about 8500–40 000 foods.^23^ However, the validity of nutrition information provided by MyFitnessPal is questionable because the extensive database is a result of the food entries contributed by the app’s end-users with no way of validating the accuracy of the energy and nutrient values.^3^

Based on a study in Brazil, dietary analysis with MyFitnessPal is accurate and efficient for total EI and macronutrients.^24^ Several studies have also shown that MyFitnessPal is a good app to use as dietary assessment tool in intervention studies related to obesity.^1 3–5^ In this study, however, Bland-Altman plots showed overall weak agreement between MyFitnessPal and FCT in estimating the energy and macronutrient intakes of Filipino adults with obesity. This can be explained again by the quality of the FCD of consumer-grade apps such as MyFitnessPal.^23^ Considering that the food databases of these apps are not based on standard Philippine database, these apps may provide inaccurate estimates of caloric and macronutrient intake of foods consumed locally. Moreover, other food entries in the database are entered by users themselves and therefore the food entries may not display correct values of some nutrients or could even show incorrect ones because the only required information to create a food entry is its name and energy content.^3^

### Intercoder reliability: comparison of energy and nutrient intake estimates using MyFitnessPal among dietitians

Apart from individuals with obesity, healthcare professionals may also benefit from nutrition apps for use in their clinical practice provided that these apps are valid and reliable.^25^ Filipino nutritionist–dietitians had not fully exploited apps in their dietary assessment practice even though smartphone technology in the Philippines is mature and widespread. This may be because reliability of MyFitnessPal among health experts is not yet established.^23^ In this study, energy and macronutrient intake estimates among dietitians suggest good intercoder reliability, with the exception of fat intake estimates. Knowing this, healthcare professionals may be more inclined to incorporate apps into their practice and/or recommend nutrition apps to patients with caution. With supplementary nutrition education and guidance in using these apps, nutrition professionals may help patients self-monitor their food intake and, consequently, in long-term self-management of obesity.

### Likelihood of misreporting energy intake

Food records can place a high burden on the respondent and risk an alteration of habitual intake^6^ leading to implausible EI reports. Implausible reported EI reduce the overall validity of a sample, and not excluding them may lead to inappropriate conclusions about potential dietary causes of health outcomes such as obesity.^26 27^ Fortunately, the prevalence of misreporting of food intake among the participants in this study is very low. This may be because the participants were asked to record their food intake for 5 days only. The participants were also instructed not to alter their food intakes and not to follow certain weight loss-related diet or exercise regimens during the study.

The Goldberg approach takes into consideration both the reported EI and the reported physical activity. This may explain that, even though their reported EI are less than 2000 calories which is surprising considering that they have obesity, the participants’ sedentary lifestyle may cause the participants’ EE to be lower than their EI leading to weight gain and obesity.

Limitations of this study include the use of self-reported measurements of height and weight by untrained individuals which may lead to inaccurate BMI and BMR calculations. Similarly, self-reported dietary intakes and PAL are prone to recall bias, portion size estimation errors and misreporting. Additionally, all participants were young adults, so the study’s results may not be generalisable to other age groups. Participants were recruited from online weight loss communities, potentially making them highly motivated to follow dietary recommendations. Some dietitians struggled with the MyFitnessPal app, citing mental fatigue and difficulties in selecting appropriate food items from the database, and noted that the app lacked certain Filipino foods. On the other hand, the Philippine FCT lacked processed and packaged foods, which are commonly consumed by participants. Lastly, the Goldberg approach used to screen implausible energy reporters has limitations, as self-reported physical activity is subject to recall and response biases.

## Conclusion

Results of this study showed MyFitnessPal to have poor construct validity and poor relative validity among Filipinos with obesity but with good intercoder reliability among dietitians. Energy and nutrient data generated using MyFitnessPal do not resemble those generated using the standard dietary assessment method. Moreover, the ability of MyFitnessPal to reflect accurate estimates of energy and macronutrient intakes of Filipino adults with obesity is questionable. Prior nutrition knowledge is a factor in ensuring the accuracy of energy and nutrient intake data generated using MyFitnessPal app. To help Filipino adults with obesity maximise the use of this app in self-monitoring their food and nutrient intake, it is recommended that they consult with a nutritionist–dietitian early on during weight management interventions for proper guidance on how to use these apps.

## Data Availability

Data are available upon reasonable request.
